# A health facility-based assessment of the ancillary benefit for prevention of anaemia at term of intermittent preventive therapy with sulfadoxine-pyrimethamine in pregnancy

**DOI:** 10.4314/gmj.v58i4.4

**Published:** 2024-12

**Authors:** Brainard A Asare, Grace Asare

**Affiliations:** 1 Ghana Health Service, Kwaebibirem Municipal Health Directorate, Kade, Eastern Region, Ghana; 2 Ghana Health Service, Kade Government Hospital, Eastern Region, Ghana

**Keywords:** Intermittent, preventive, therapy, maternal, anaemia, benefit, pregnancy

## Abstract

**Objective:**

This study aims to evaluate the ancillary benefit of intermittent preventive therapy with sulfadoxine-pyrimethamine (IPTp-SP) in preventing maternal anaemia (MA) among parturient women differentially exposed to the regimen.

**Design:**

A health facility-based retrospective analytical cross-sectional study.

**Settings:**

The study was conducted at the Kade Government Hospital's maternity/labor suit.

**Participants:**

Data from 2,545 parturient women were abstracted from birth registers.

**Statistical analysis:**

Baseline characteristics were described, and stratified analyses assessed their impacts. Differences in mean mHgbc based on IPTp-SP exposure were determined using one-way ANOVA. An unpaired two-sample t-test evaluated the significance of inter-dose group differences. The bivariable analysis examined crude and adjusted risks of anaemia with differential IPTp-SP exposure.

**Main outcome measure:**

The main outcome measure was the level of mHgbc with varying IPTp-SP exposure.

**Results:**

The overall mean exposure to IPTp-SP was 2.35 (±2.35) doses. Of the women, 5.9% had ‘nil’ exposure, with a mean mHgbc of 9.71 g/dL (±1.6). Among the 75.9% who received 1-3 doses, the mean mHgbc was 10.39 g/dL (±1.3). For the 18.2% who received ≥4 doses, the mean mHgbc was 10.77 g/dL (±1.4). The mHgbc notably rose as the mean doses of IPTp-SP increased. The crude odds ratios (COR) were 1.96 (95% CI: 0.99-3.89, p = 0.06) for ‘nil’ exposure, 1.28 (95% CI: 0.92-1.78, p = 0.16) for 1-3 doses, and 0.59 (95% CI: 0.41-0.84, p = 0.002) for ≥4 doses.

**Conclusion:**

The consistent linear increase in mean mHgbc with higher IPTp-SP doses remains clinically crucial.

**Funding:**

None declared

## Introduction

Intermittent preventive therapy (IPT) is a public health intervention aimed at mitigating and managing malaria episodes in susceptible populations, including infants (IPTi), children (IPTc), schoolchildren (IPTsc), and pregnant women (IPTp). IPT combines two well-established strategies for malaria control: eradicating existing parasites (similar to mass drug administration) and prophylactic measures to prevent new infections. Sulfadoxine-pyrimethamine SP) is the current medication used for IPTp-SP to achieve these objectives.^1^ The groundbreaking implementation of IPTi-SP was initiated in Ifakara, Tanzania, in 1999, resulting in a substantial 59% reduction (95% CI, 41%-72%) in clinical malaria episodes.^2^-^6^ Similarly, in Senegal, where malaria exhibits a pronounced seasonal pattern, SP with artesunate for IPTc consistently demonstrated a remarkable 86% reduction (95% CI 80-90) in malaria attacks across multiple malaria seasons.^7^ A study conducted in Kenya demonstrated that implementing IPTsc using SP and amodiaquine resulted in a substantial decrease in the prevalence of anaemia.^8^ IPTp involves the administration of a therapeutically effective dosage of an antimalarial drug at least twice during pregnancy, regardless of malaria infection status. This intervention, implemented within the framework of ‘Directly Observed Therapy [DOT]’ at the antenatal clinic, is recommended by the World Health Organization (WHO) due to its proven safety and effectiveness during pregnancy.^9^

Several studies have demonstrated the high efficacy of IPTp-SP in comparison to placebo or chloroquine prophylaxis for preventing placental infection, low birth weight (LBW), and/or severe maternal anaemia (MA).^10^

Emerging evidence in Tanzania, however, suggests that IPTp-SP may no longer be effective. Pertinent concerns point to the use of partially effective anti-malarial agents for IPTp, leading to exacerbated malaria infections in settings of widespread drug resistance.^14^ Conflicting reports on efficacy in preventing malaria, MA, hospital admissions, and maternal mortality have engendered valid debates on its widespread implementation.^15^ Besides its primary objective of preventing malaria during pregnancy, the effectiveness of IPTp-SP in preventing adverse consequences on maternal and fetal outcomes, such as MA, fetal anaemia, LBW, and neonatal mortality, is crucial.^16^,^17^ MA underlies malaria's significant contribution to maternal deaths. However, the importance of its management may sometimes elude certain healthcare providers.^18^ A study found that 18% of pregnant women with severe MA had an active malaria infection without peripheral blood parasites, compared to 7% of parturient women with higher mHgb concentrations (mHgbc).^19^

Malaria is causally linked to up to 34% of severe MA in parturient women admitted to deliver in hospitals, with over 50% of these cases lacking a positive peripheral blood film or malaria parasitemia-associated febrility.^19^ Malaria-associated hemolysis and bone marrow dyserythropoiesis underlie the low Hgbc in infants, young children, and adults in high transmission areas.^20^

Research to comprehend the relationship between malaria and anaemia is arduous in high-transmission settings due to nonspecific parasitological diagnosis and the widespread practice of self-medication for febrile illnesses, among others.^21^ Frequent concomitant haemoglobinopathies, nutritional deficiencies (especially iron deficiency), and intestinal helminthiasis further compound the arduousness.^21^, ^22^ Malaria (at all transmission levels) significantly contributes to MA and poor birth outcomes.^23^ Falciparum malaria, which is directly linked to maternal mortality in low-transmission settings, indirectly contributes to maternal mortality in high-transmission settings, primarily by increasing the risk of MA.^24^ Recurrent infection from anti-malarial drug resistance may further exacerbate malaria-associated anaemia.^25^ Pronounced associations between malaria infection and MA among primigravidae are evident.^26^ Evidence in Ghana (where malaria accounts for 13.8% of outpatient department visits, 10.6% of admissions, and 9.4% of maternal mortality) suggests that preventive chemoprophylaxis during pregnancy decreases infection incidence, elevates mHgbc, and prevents LBW births.^26^ Consequently, Ghana has implemented the recommended standard of care for malaria in high-prevalence settings during pregnancy (per WHO specifications), involving the provision of IPTp-SP.^26^

Analyses of its prophylactic impact post-implementation remain limited amidst reports on growing SP resistance in West Africa.^24^, ^26^ This study aims to evaluate the ancillary benefit of IPTp-SP in preventing MA among parturient women differentially exposed to the regimen.

## Methods

A health facility-based retrospective analytical cross-sectional method was employed to examine obstetric care service data extracted from birth registers and other relevant sources at the maternity/labour suit of Kade Government Hospital. The hospital, the only referral facility in Kwaebibirem municipality, provides secondary healthcare to about 146,346 residents, including approximately 35,123 women of reproductive age. Variables were included in the study through a retrospective review of all obstetric health records. The study used non-probability convenience sampling of available records on ANC attendant parturient women who delivered at Kade Government Hospital between 2019 and 2022.

The mHgbc level, recorded in the birth register and considered a significant and accessible measure at the district health facility level, was strictly used to define MA status. The classification of MA was based on the existing range, which defines it as mHgbc of less than 11.0 g/dL in healthy women supplemented with iron before delivery.^27^ Our research assumed that all parturient women did not have hemoglobinopathies. The severity of MA was classified based on mHgbc levels as follows: severe anaemia ≤ 6.9 g/dL, moderate anaemia 7.0-9.9 g/dL, and mild anaemia 10.0-10.9 g/dL. Exposure levels to IPTp-SP were analysed using a trichotomised approach: ‘nil’ exposure (no doses received throughout pregnancy), exposure to 1-3 doses, and exposure to ≥4 doses. Pre-delivery mHgbc, routinely checked during in-patient obstetric care, exclusively served as reference values for classifying MA. Post-delivery mHgbc were not used to avoid potential fluctuations.

The birth register, serving as the study's primary data source, was partitioned into four distinct segments to improve data abstraction. The initial variables acquired from the first part of the register included personal information, i.e., age, residential classification (urban/peri-urban or rural), highest education level attained, gravidity, and parity. ANC indicators, such as mHgbc, ANC attendance, gestational age (GA) at birth, IPTp-SP doses during pregnancy, and maternal ABO phenotypic blood groups, comprised the second set of variables from the second section. This section additionally documents information on maternal syphilis, hepatitis B and HIV infection status, as well as maternal systolic and diastolic blood pressures (or SBP and DBP).

The third section, generally indicating neonatal health at birth, records the APGAR score, fetal heart rate, fetal respiration within 30 minutes, fetal presentation, and measures of fetal dimensions. The fourth section records data on post-delivery complications, e.g., postpartum haemorrhage (PPH), antepartum haemorrhage (APH), and obstructed labour, among others. The categorisation of urban/peri-urban and rural communities was consistent with the threshold population sizes used to define these areas. Specifically, the Ghana Statistical Service defines urban areas as localities with a population of at least 5,000 since 1960. Peri-urban communities refer to those located adjacent to urban areas whose social dynamics are similar to those of the adjoining urban area.

The variables ‘married’ and ‘cohabiting’ represented one variable analysis level due to the perceived similarity in their dynamics. The pre-birth mHgbc served as the baseline. Since the secondary data only included one mHgbc reading per subject, this was handled as the primary measurement. There is no separate end-line measurement. We compared pre-birth mHgbc levels across different IPTp-SP exposure levels (‘nil’, 1-3 doses, and ≥4 doses) to assess the impact on MA. Ethical approval for the study was obtained from the Ghana Health Service Ethics Review Committee (GHS-ERC: 031/10/23). The Ethics Review Committee approved surrogate consent from the hospital's medical superintendent, head of nursing, and nurse manager for the study. They were authorised to sign consent forms on behalf of parturient women, considering non-traceability in the secondary data. This approval also allowed the design of an information sheet for the hospital management team to sign surrogate consent.

### Statistical analysis

The baseline characteristics of parturient women were descriptively analysed based on mean IPTp-SP exposure during pregnancy and the corresponding mean mHgbc. Stratified analyses evaluated the proportional contributions of each characteristic, presented as mean estimates with standard deviations (SD). The normality of mHgbc values was assessed using the Shapiro-Wilk test, which yielded a p-value of 0.069 (W = 0.97), indicating no significant deviation from normality. Consequently, oneway ANOVA was used to evaluate differences in mean mHgbc across three IPTp-SP exposure levels. A total of 108 mHgbc values exhibited a mean of 9.3 (±3.36) and a median of 9.55, with skewness (-0.44) indicating slight leftward skew and kurtosis (-0.26) suggesting a mesokurtic distribution. No significant outliers were detected. The F-statistic and its associated p-value determined differences among the exposure levels. Pairwise comparisons (e.g., ‘nil’ vs. 1–3 doses, and 1–3 vs. ≥4 doses) were performed using unpaired two-sample t-tests with Bonferroni correction (adjusted α′ = 0.01) to minimise Type-I error.

The risk of MA was evaluated using crude bivariable estimates and subgroup-adjusted Mantel-Haenszel odds ratios with corresponding confidence intervals (CIs). A chi-squared test for interaction assessed subgroup differences. Initial analyses treated variables as continuous and normally distributed; subsequently, variables were recoded into categorical forms and analysed in bivariable models. All statistical analyses were conducted using OpenEpi and Epi Info version 3.5.1.

## Results

Data on 2,545 parturient women were abstracted from birth registers, excluding 28 non-ANC attendants. These exclusions, considered an insignificant minority, were assumed not to impact the analysis. Exposure to IPTp-SP varied, with pregnant women receiving between 1 and 7 doses. About 94.1% of parturient women received at least one dose of IPTp-SP, with an overall mean exposure of 2.35 (±2.35) doses among those who received at least one dose before delivery. In distribution terms, 5.9% of women had nil exposure, with a mean mHgbc of 9.71 g/dL (±1.6). Among the 75.9% of women who received 1-3 doses, the mean mHgbc was 10.39 g/dL (±1.3). For the 18.2% who received ≥4 doses, the mean mHgbc was 10.77 g/dL (±1.4). The mHgbc ranged from 1.6 to 16.3 g/dL, increasing from MA to normal levels. The overall mean mHgbc was 10.29 g/dL (±1.4), exhibiting a tendency to rise with increasing mean doses of IPTp-SP (Table 1).

**Table 1:** Baseline characteristics of parturient women and analysis of mean IPTp-SP exposure and maternal haemoglobin concentration

Characteristics	Characteristic – N (%)	Mean IPTp-SP (±SD) Doses	Mean (±SD) maternal hemoglobin concentration
**Age groups**			
≤20 years	553 (21.8)	1.83 (1.16)	9.93 (1.52)
21-30 years	1215 (48.0)	2.50 (1.26)	10.50 (3.27)
31-40 years	689 (27.2)	2.46 (1.23)	10.40 (1.37)
≥41 years	74 (3.0)	2.42 (1.02)	10.35 (1.40)
**Residence Area**			
Urban	1285 (52)	2.47 (1.31)	10.37 (1.43)
Rural	1187 (48)	2.22 (1.17)	10.33 (3.28)
**Highest education attained**			
≤Junior high	1942 (78.2)	2.27 (1.22)	10.31 (2.75)
Senior high	403 (16.2)	2.37 (1.21)	10.23 (1.47)
Tertiary	137 (5.5)	3.37 (1.34)	11.08 (1.19)
**Occupation**			
Formal	232 (10.6)	2.78 (1.39)	10.63 (1.37)
Non-formal	1952 (89.4)	2.35 (1.23)	10.38 (2.73)
**Parity**			
Para 1	781 (31.0)	2.34 (1.30)	10.14 (1.54)
Para 2-4	1558 (61.8)	2.39 (1.20)	10.46 (2.96)
≥Para 5	184 (7.3)	2.22 (1.14)	10.21 (1.37)
**Prenatal care attendance**			
Yes	1594 (81.9)	2.36 (1.25)	10.28 (1.41)
No	353 (18.1)	2.15 (1.02)	10.57 (6.37)
**Term status**			
Preterm	205 (8.7)	2.04 (1.15)	10.10 (1.78)
Early-term	569 (24.1)	2.28 (1.31)	10.34 (4.50)
Full-term	1132 (47.9)	2.37 (1.27)	10.39 (1.36)
≥Late-term	458 (19.4)	2.54 (1.18)	10.41 (1.46)
**ABO phenotypic blood group**			
Group A	429 (21.3)	2.33 (1.17)	10.27 (1.42)
Group B	67 (3.3)	2.59 (1.26)	10.25 (1.66)
Group C	376 (18.7)	2.51 (1.27)	10.34 (1.44)
Group D	1138 (56.6)	2.36 (1.28)	10.43 (3.23)
**Syphilis status**			
Positive	56 (2.3)	2.35 (1.49)	10.26 (1.50)
Negative	2352 (96.3)	2.36 (1.24)	10.37 (2.55)
Not tested	35 (1.4)	1.83 (1.16)	9.49 (1.80)
**Hepatitis B**			
Positive	107 (4.4)	2.41 (1.29)	10.46 (1.53)
Negative	2302 (94.3)	2.36 (1.24)	10.36 (2.57)
Not tested	32 (1.3)	1.87 (1.12)	9.74 (1.85)
**HIV status**			
Positive	50 (2.1)	2.22 (0.87)	10.03 (1.83)
Negative	2329 (96.6)	2.36 (1.26)	10.38 (2.54)
Not tested	31 (1.3)	2.87 (1.35)	9.01 (2.43)
**Systolic blood pressure**			
<130 mmHg	1783 (76.9)	2.35 (1.24)	10.24 (1.46)
130-139 mmHg	251 (10.8)	2.37 (1.32)	11.03 (6.32)
≥140 mmHg	286 (12.3)	2.46 (1.30)	10.44 (1.43
**Diastolic blood pressure**			
<80 mmHg	1501 (64.9)	2.33 (1.26)	10.22 (1.42)
80-89 mmHg	516 (22.3)	2.43 (1.21)	10.29 (1.44)
≥90 mmHg	279 (12.8)	2.43 (1.31)	11.13 (6.01)

The one-way ANOVA analysis of variance in mean mHgbc across trichotomized IPTp-SP exposure levels revealed a trend of increasing mean mHgbc with higher IPTp-SP doses. Significant variations were observed among the parturient women's specific characteristics (Table 2).

**Table 2:** Exploration of mean maternal haemoglobin concentrations across three IPTp-SP exposure groups by maternal and pregnancy characteristics

Characteristics	Mean (±SD) haemoglobin concentration			ANOVA	
	Nil IPTp-SP doses	1-3 IPTp-SP doses	≥4 IPTp-SP doses	F statistic	p-value
**Age groups**					
≤20 years	9.49	10.07	10.60	2.44	0.08
21-30 years	10.57	10.42	10.81	8.90	0.0002
31-40 years	9.28	10.49	10.80	5.73	0.0038
≥41 years	0.0	11.00	9.86	1.83	0.08
**Residence Area**					
Urban	9.80	10.39	10.84	6.92	0.001
Rural	12.96	10.40	10.66	3.92	0.02
**Highest education attained**					
≤Junior high	11.71	10.42	10.70	2.53	0.08
Senior high	9.41	10.12	10.58	2.07	0.12
Tertiary	10.80	11.20	11.21	0.17	0.84
**Occupation**					
Formal sector	11.05	10.32	11.18	3.95	0.02
Non-formal sector	11.92	10.47	10.64	3.07	0.04
**Parity**					
Para 1	9.67	10.19	10.73	4.16	0.01
Para 2-4	12.96	10.48	10.81	5.03	0.006
≥Para 5	9.30	10.36	10.54	0.70	0.50
**Prenatal care**					
Yes	9.77	10.35	10.68	6.18	0.002
No	8.10	10.20	10.82	1.82	0.17
**Term status**					
Preterm	9.80	10.22	10.61	0.29	0.74
Early-term	9.69	10.26	10.42	1.26	0.28
Full-term	9.88	10.42	10.85	4.99	0.007
≥Late-term	9.45	10.56	11.00	5.41	0.005
**Maternal ABO phenotypic blood groups**					
Group A	10.26	10.46	10.63	0.36	0.69
Group AB	7.20	10.49	10.50	4.62	0.01
Group B	10.46	10.47	10.89	1.05	0.35
Group O	12.57	10.38	10.90	3.61	0.02
**Syphilis status**					
Positive	7.00	10.73	10.76	3.03	0.08
Negative	11.61	10.38	10.78	3.69	0.02
Not tested	6.20	9.72	0.0	9.23	0.05
**Hepatitis B**					
Positive	9.73	10.46	11.38	1.96	0.15
Negative	11.63	10.39	10.74	3.39	0.03
Not tested	6.20	10.10	0.0	6.18	0.06
**HIV status**					
Positive	7.40	10.01	0.0	2.31	0.15
Negative	11.60	10.40	10.83	3.66	0.02
Not tested	6.20	9.90	8.10	0.21	0.81
**Systolic blood pressure**					
<130 mmHg	9.80	10.33	10.84	8.92	0.002
130-139 mmHg	10.00	10.65	10.70	0.81	0.44
≥140 mmHg	9.20	10.54	10.42	1.88	0.15
**Diastolic blood pressure**					
<80 mmHg	9.64	10.29	10.93	13.24	0.00001
80-89 mmHg	10.78	10.40	10.45	0.16	0.84
≥90 mmHg	9.73	10.84	10.54	1.98	0.14

An unpaired two-sample t-test investigated findings from the one-way ANOVA, focusing on inter-dose group mean mHgbc variations with significant F-statistics. Significant differences were found in age (21-30 years), formal occupation, urban residence, uniparous status, fullterm status, and SBP <130 mmHg between 1-3 and ≥4 doses. Significant inter-dose group mean mHgbc variations were also noted in age (≥31 years), rural residence, junior high school education, non-formal occupation, multiparity, birth at >40 weeks, blood group AB, and DBP ≥90 mmHg between ‘nil’ and 1-3 doses. Significant differences were observed in blood group O and DBP <80 mmHg between ‘nil’ and 1-3 doses and between 1-3 and ≥4 doses, Table 3.

**Table 3:** Analysis of significance of inter-dose group mHgb concentration variations by maternal and pregnancy characteristics using the unpaired two-sample t-test

Characteristic	‘Nil’ vs 1-3 IPTp-SP doses			1- 3 vs ≥4 IPTp-SP doses		
	t-test	df	p-value	t-test	df	p-value
Age groups						
**≤20 years**	1.62	148	0.10	1.23	139	0.21
**21-30 years**	0.64	323	0.51	3.53	113	0.0006
**≥31 years**	2.96	188	0.0034	0.99	225	0.32
Residence Area						
**Urban**	2.07	325	0.039	2.77	401	0.0058
**Rural**	2.61	324	0.0093	0.35	49.04	0.72
Highest level of education attained						
**≤Junior high**	3.14	510	0.0018	1.81	565	0.07
**Senior high**	1.44	125	0.15	1.50	149	0.13
**Tertiary**	0.52	19	0.60	0.028	44	0.97
Occupation						
**Formal sector**	0.64	47	0.52	2.82	77	0.0060
**Non-formal sector**	3.07	519	0.0022	0.34	108	0.73
Parity						
**Para 1**	2.06	371	0.039	2.40	428	0.016
**Para 2-4**	2.18	239	0.010	1.89	275	0.059
**≥Para 5**	1.05	51	0.29	0.38	58	0.70
Term status at birth						
**Preterm/Early term**	0.45	49	0.65	0.54	52	0.58
**Full-term**	1.85	298	0.065	2.42	345	0.015
**≥Late-term**	2.47	143	0.014	1.79	172	0.07
Maternal ABO phenotypic blood groups						
**Group A**	0.39	117	0.69	0.69	140	0.48
**Group AB**	2.86	20	0.0097	0.015	26	0.98
**Group B**	0.039	120	0.96	1.47	141	0.14
**Group O**	3.13	339	0.0019	3.05	390	0.0024
Systolic blood pressure						
**<130 mmHg**	2.16	481	0.03	3.34	554	0.0009
**130-139 mmHg**	1.18	75	0.23	0.15	86	0.87
**≥140 mmHg**	1.87	66	0.065	0.36	84	0.71
Diastolic blood pressure						
**<80 mmHg**	2.62	402	0.0090	4.07	458	0.0001
**80-89 mmHg**	0.95	135	0.34	0.82	159	0.41
**≥90 mmHg**	1.84	84	0.068	0.98	106	0.32

MA burden decreased with higher IPTp-SP doses, as shown by decreasing crude odds ratios (COR) of 1.96 (95% CI: 0.99-3.89, p = 0.06) for no ‘nil’ exposure, 1.28 (95% CI: 0.92-1.78, p = 0.16) for 1-3 doses, and 0.59 (95% CI: 0.41-0.84, p = 0.002) for ≥4 doses. These associations, adjusted for maternal and pregnancy characteristics within subgroups, showed insignificant chi-squared interaction tests, suggesting no significant differences across subgroups (Table 4).

**Table 4:** Bivariable analysis of maternal hemoglobin concentration relative to IPTp-SP dose exposure and subgroup characteristics

Characteristic	‘Nil’ vs ≥1 IPTp-SP doses		0-3 vs ≥4 IPTp-SP doses		≥4 vs ≤3 doses	
	MHAOR (CI)	p-value (interaction)	MHAOR (CI)	p-value (interaction)	MHAOR (CI)	p-value (interaction)
Age groups						
**Reference: ≤20 years**	1.71	0.05	1.26	1.55	0.62	0.54
**Comparison: ≥21 years**	(0.86-0.34)		(0.90-1.79)		(0.43-0.90)	
**Reference: 21-30 years**	1.89	0.10	1.27	2.93	0.60	1.31
**Comparison: <20 and >30 years**	(0.95-3.75)		(0.91-1.72)		(0.41-0.86)	
**Reference: ≥31 years**	1.94	0.04	1.28	0.97	0.59	1.71
**Comparison: ≤30 years**	(0.98-3.85)		(0.92-1.78)		(0.41-0.84)	
Residence Area						
**Reference: Urban**	1.96	0.26	1.26	0.83	0.59	0.88
**Comparison: Rural**	(0.99-3.88)		(0.90-1.76)		(0.41-0.85)	
**Reference: Rural**	1.96	0.43	1.26	1.14	0.59	1.29
**Comparison: Urban**	(0.99-3.87)		(0.90-1.76)		(0.41-0.85)	
Highest education attained						
**Reference: ≤Junior high**	1.96	1.98	1.29	9.10	0.57	4.54
**Comparison: Senior high & Tertiary**	(0.99-3.89)		(0.92-1.80)		(0.40-0.83)	
**Reference: Senior high**	1.98	3.32	1.27	2.93	0.58	0.64
**Comparison: Junior high & Tertiary**	(1.01-3.90)		(0.91-1.77)		(0.40-0.84)	
**Reference: Tertiary**	1.97	0.13	1.12	0.04	0.67	0.03
**Comparison: Junior high & Senior high**	(0.99-3.92		(0.79-1.58)		(0.46-0.98)	
Occupation						
**Reference: Formal**	1.91	0.42	1.23	2.34	0.61	0.77
**Comparison: Non-formal**	(0.96-3.79)		(0.88-1.72)		(0.42-0.89)	
**Reference: Non-formal**	1.96	0.64	1.27	2.64	0.59	4.18
**Comparison: Formal**	(0.99-3.89)		(0.91-1.78)		(0.41-0.84)	
Parity						
**Reference: Para 1**	1.98	0.42	1.28	0.24	0.58	0.12
**Comparison: Para 2-4 & ≥Para 5**	(1.00-3.92)		(0.92-1.79)		(0.40-0.83)	
**Reference: Para 2-4**	1.97	1.23	1.29	1.45	0.58	0.74
**Comparison: Para 1 & ≥Para 5**	(0.99-3.90)		(0.92-1.79)		(0.40-0.84)	
**Reference: ≥Para 5**	1.97 (0.99-	1.06	1.28	1.85	0.58 (0.40-0.83)	0.74
**Comparison: Para 1-4**	3.90)		(0.92-1.79)			
Term status at birth						
**Reference: Pre-/Early-term**	1.91	0.09	1.28	0.01	0.59	0.01
**Comparison: Full-term & ≥Late-term**	(0.96-3.79)		(0.92-1.79)		(0.41-0.85)	
**Reference: Full-term**	1.94	0.10	1.28	0.47	0.59	0.23
**Comparison: Pre-/Early-term & ≥Late-term**	(0.98-3.86)		(0.92-1.79)		(0.41-0.84)	
**Reference: ≥Late-term**	1.94	1.13	1.28	1.31	0.59	0.22
**Comparison: Pre-/Early term & Full-term**	(0.98-3.86)		(0.92-1.79)		(0.41-0.85)	
Maternal ABO phenotypic blood groups						
**Reference: Group A**	1.93	0.004	1.32	0.02	0.58	0.001
**Comparison: Group AB, B & O**	(0.93-3.98)		(0.94-1.87)		(0.40-0.84)	
**Reference: Group AB**	1.88	0.10	1.32	0.56	0.58	0.08
**Comparison: Group A, B & O**	(0.91-3.86)		(0.94-1.86)		(0.40-0.85)	
**Reference: Group B**	1.89	2.04	1.33	0.01	0.58	0.74
**Comparison: A, AB & O**	(0.91-3.91)		(0.94-1.87)		(0.40-0.84)	
**Reference: Group O**	1.88	0.73	1.33	0.03	0.58	0.68
**Comparison: A, AB, & B**	(0.91-3.87)		(0.94-1.87)		(0.40-0.84)	
Systolic blood pressure						
**Reference: <130 mmHg**	1.95	0.12	1.30	4.91	0.59	5.43
**Comparison: 130-139 mmHg & ≥140 mmHg**	(0.95-3.98)		(0.93-1.82)		(0.41-0.85)	
**Reference: 130-139 mmHg**	1.96	0.35	1.31	0.32	0.58	1.48
**Comparison: <130 mmHg & ≥140 mmHg**	(0.96-4.03)		(0.93-1.83)		(0.40-0.84)	
**Reference: ≥140 mmHg**	1.94	0.12	1.30	0.13	0.59	3.28
**Comparison: <130 mmHg & 130-139 mmHg**	(0.95-3.97)		(0.93-1.82)		(0.41-0.85)	
Diastolic blood pressure						
**Reference: <80 mmHg**	1.90	0.06	1.31	1.32	0.59	2.59
**Comparison: 80-89 mmHg & ≥90 mmHg**	(0.93-3.90)		(0.93-1.84)		(0.41-0.85)	
**Reference: 80-89 mmHg**	2.26	0.44	1.20	1.02	0.58	0.01
**Comparison: <80 mmHg & ≥90 mmHg**	(1.03-4.99)		(0.83-1.75)		(0.40-0.84)	
**Reference: ≥90 mmHg**	1.93	0.66	1.27	1.51	0.60	4.53
**Comparison: 80-89 mmHg & 80-89 mmHg**	(0.94-3.95)		(0.91-1.78)		(0.42-0.87)	

## Discussion

This study investigated the beneficial ancillary effect of IPTp-SP in preventing MA under the assumption that parturient women did not have hemoglobinopathies. The descriptive data analysis revealed inconsistencies in observations, particularly in variables like age, residence area, occupation status, and blood group, which were not consistently recorded in the secondary data. Since this data was not originally collected for this research, it was impossible to follow up and fill in the missing fields.

The mean IPTp-SP exposure throughout pregnancy varied from 1.83 (±1.16) among adolescents and some parturient women not tested for syphilis during pregnancy to 2.47 (±1.31) among urban residents, corresponding with lower and higher mean mHgbc, respectively. Most parturient women received 1-3 doses throughout pregnancy, with an overall mean of 2.35 (±2.35) doses, below the WHO recommended minimum of three doses. Four or more doses represented a minority. The investigation of the reasons for ‘nil’ exposure was beyond the scope of this study. This study's overall IPTp-SP coverage of 94.1%, defined by varied exposure levels, was higher than a study that reported 85.7%.^28^ Exploration of this phenomenon was beyond the scope of this investigation, as it solely relied on secondary data not originally collected for research purposes. This approach provides primarily an analytical cross-sectional perspective, lacking explanatory factors that underpin the observed phenomena.

As institutional data indicates, Ghana recorded increased IPTp-SP coverage between 2014 and 2017. The nationwide coverage exhibited a substantial increase, rising from 30.0% to 74.0%, with the Greater Accra region demonstrating notable improvement from 32.4% to 79.0% over the identical temporal span.^29^ A hospitalbased cross-sectional study aimed to estimate the prevalence of MA and its associated factors. The study reported a drop in prevalence from the first ANC visit to the current visit, i.e., from 34.5% to 28.4%.^30^ A 6.1% drop in the level of MA was attributed to ANC services, which include the provision of insecticide-treated nets (ITN), iron supplementation, malaria prophylaxis, and counselling, among other interventions, provided to pregnant women.^30^ The overall estimated prevalence of MA in the current study (68.1%) is higher than that reported in other studies conducted in Ghana.

Specifically, previous studies found a prevalence of 34.4% in Sekondi-Takoradi, 50.8% in Tamale Teaching Hospital in Northern Ghana, 56.5% in the Ashanti Region, and 57.1% in Sekyere West in southern Ghana.^31^-^33^ This high MA prevalence, however, compares with the 70.0% in twenty-five rural communities in Northern Ghana.^32^

Compared to similar studies conducted in various regions of Africa and other parts of the world, the prevalence of MA in this study was higher than that reported in the following locations: 54.5% in Nigeria, 42.7% in South Africa, 51.3% in rural Egypt, 57.0% in Kenya, 57.7% in Lahore, Pakistan, and 59.0% in India.^34^-^37^ A WHO report indicates that 62.0% of pregnant women in Ghana have mHgbc below 11.0 g/dL, a level considered a public health concern.^38^ In this study, despite an overall mean mHgbc of 10.2 g/dL, indicative of MA, around 29.0% of parturient women exhibited mHgbc above 11.0 g/dL. Notably, the strata-specific mean mHgbc across characteristics remained largely consistent with MA. Parturient women in this study with lower IPTp-SP exposure showed consistently lower mean mHgbc. This finding was validated through bivariable analysis, demonstrating that receiving ≥4 doses significantly reduced the likelihood of MA. These results logically support the hypothesised ancillary benefit of IPTp-SP in preventing MA, suggesting a potential impact of the independent variable (doses of IPTp-SP) on the dependent variable (mHgbc).

While statistically insignificant variations in mean mHgbc were observed among the three IPTp-SP exposure groups in the preferred data analysis approaches, this study underscores the critical significance of a sustained and consistent trend of higher mean mHgbc with increased IPTp-SP doses. We considered this observation internally aligned, albeit debatably, with Hill's criterion of causality, especially consistency, highlighting the notable linear trend of higher mean mHgbc with increased IPTp-SP doses.^39^ This pattern persisted across stratumspecific risk estimates (expressed as MHAORs), indicating a reduced risk of MA with exposure to ≥4 doses of IPTp-SP during pregnancy. The effect was consistent across all subgroups, with the most pronounced protection benefits in the ‘≥4 vs ≤3 doses’ exposure subgroup. The lowest MHAORs were observed in: Senior high school - 0.57 (0.40-0.83), p-value 4.54; Tertiary education - 0.58 (0.40-0.84); Pre-/Early term - 0.59 (0.410.85); phenotypic ABO blood group A - 0.58 (0.40-0.84); and SBP <130 mmHg - 0.59 (0.41-0.85). Multiple IPTp-SP doses, therefore, possibly predict lower MA burdens across various demographics.^16^

In partial concordance with this study's findings, a systematic review and meta-analysis on IPTp-SP for malaria during pregnancy found that administering ≥3 doses to primigravidae and secundigravidae in Africa decreased the risk of moderate to severe MA and LBW.^40^ This observation is consistent with the 2012 WHO revision of the IPTp-SP regimen, which advocates for a minimum of three doses to enhance protection against malaria and related MA. A cross-sectional study reported an overall MA prevalence of 62.6%, with a lower prevalence of 54.1% among women exposed to ≥3 doses, compared to 66.6% among those exposed to two doses.^41^ A cross-sectional study assessing the efficacy of IPTp-SP in preventing malaria and MA among parturient women attending ANC definitively asserted that the demonstrated effectiveness of the IPTp-SP regimen in preventing both malaria and MA holds significant promise for alleviating the burden of malaria and MA in Ghana.^26^ An investigation into the utilisation of IPTp-SP through a community-based delivery system and its impact on parasitemia, MA, and LBW in Uganda revealed a substantial reduction in the prevalence of severe MA associated with the use of IPTp-SP.^42^

Despite drug resistance reducing its effect on parasitemia, community-based approaches increase access and adherence to IPTp-SP, impacting MA, parasitemia, and LBW.^42^ An improved strategy to prevent MA is crucial, as it contributes to an estimated 18% of perinatal mortality and 20% of maternal mortality in South Asia, according to a recent meta-analysis.^42^ The precise threshold of mHgbc crucial for preventing maternal mortality remains elusive.^44^ According to the Child Health Epidemiology Reference Group (CHERG), the risk of maternal mortality significantly decreases with every 1 g/dL rise in mHgbc, although this association becomes less clear at levels above 8–9 g/dL.^43^ This study acknowledges its limitations, including reliance on a single health facility (raising generalizability concerns) and using non-probability convenience sampling. Given the research objectives, constraints, and context, this approach was deemed most feasible, making convenience sampling the most efficient for data gathering. The mHgbc measurements included pre-birth mHgb levels. Monitoring their variation throughout pregnancy with ameliorative interventions was not feasible due to secondary data not originally collected for research. However, the insights gained provide valuable information relevant to the research question.

## Conclusion

This study explored IPTp-SP's role in preventing MA alongside its limitations. The mean IPTp-SP exposure during pregnancy was 2.35 (±2.35), below the WHO-recommended minimum of three doses, with most women receiving 1-3 doses. MA prevalence (68.1%) exceeded rates in other Ghanaian studies. Women with more than 2.5 doses had consistently higher mean mHgbc, supporting WHO's recommendation of ≥3 doses to mitigate malaria's impact on maternal and fetal health. The study underscores IPTp-SP's serendipitous MA prevention benefit, stressing the need for enhanced coverage and more effective preventive strategies to improve maternal and perinatal health.
